# New molecular pieces in the jigsaw of extracellular vesicle biogenesis

**DOI:** 10.1093/plphys/kiag124

**Published:** 2026-03-09

**Authors:** Josephine H R Maidment, Marcella Teixeira

**Affiliations:** Assistant Features Editor, Plant Physiology, American Society of Plant Biologists, MD 20855, United States; PHIM Plant Health Institute, Université de Montpellier, INRAE, CIRAD, Institut Agro, IRD, Montpellier 34980, France; Centre de Biologie Structurale, INSERM, CNRS, Université de Montpellier, Montpellier 34090, France; Assistant Features Editor, Plant Physiology, American Society of Plant Biologists, MD 20855, United States; Department of Plant Pathology, Washington State University, Pullman, WA 99163, United States

Extracellular vesicles (EVs) are small, protective compartments containing diverse molecules, including proteins and nucleic acids, encapsulated by a lipid envelope. They have been observed in all 3 domains of life and are involved in distinct processes, from stress response to lateral gene transfer (Gill et al. 2019). Three basic classes of EVs have been described in mammalian cells (Jeppesen et al. 2023). Exosomes are one of these classes, originating from inward membrane budding to generate intraluminal vesicles (ILVs) within a multivesicular body (MVB). These ILVs are sorted by a specific machinery known as the endosomal sorting complex required for transport (ESCRT). Following sorting and loading, EVs rely on Rab GTPases to be trafficked to the plasma membrane, where the EVs can be secreted (Jeppesen et al. 2023). Plants, like mammals, secrete EVs. Besides MVBs, plants can form exocyst-positive organelles that are also secreted after fusion with the plasma membrane, but as a single intraluminal vesicle (Lin et al. 2015). The secretion process from the plasma membrane might involve direct budding, since plants express ANNEXIN orthologs and ANN1 and ANN2 have been observed in EV proteomes from plants (Koch et al. 2025). Specific subpopulations of EVs are secreted during biotic stress responses in plants. For example, while PATTELIN1 (PATL1) is present in distinct EVs and does not respond to stress, TETRASPANIN8 (TET8), PENETRATION1 (PEN1), and RPM1-INTERACTING PROTEIN 4 (RIN4) are present in EVs highly secreted in response to fungal infection (Koch et al. 2025). In fact, EVs have been shown to have a role in plant responses to infection by different pathogens, such as *Pseudomonas syringae* (Rutter and Innes 2017) and *Botrytis cinerea* (Wang et al. 2024).

In a recent study published in *Plant Physiology*, Koch et al. (2026) provide novel insights into the secretion of plant EVs, with a particular focus on the subpopulations of EVs marked by TET8 (TET8+) or PEN1 (PEN1+). To identify proteins involved in the biogenesis and secretion of these subpopulations of EVs, the authors performed proximity labeling experiments in *Nicotiana benthamiana*. Arabidopsis TET8 and PEN1 were fused to biotin ligase, which biotinylates proteins in proximity to the fusion protein. After verifying that the transiently expressed fusion proteins were directed to EVs in *N. benthamiana*, the authors affinity-purified biotinylated proteins for identification by MS. A small number of proteins were identified as candidate interactors for both PEN1 and TET8, including Rab GTPases, RIN4, and components of the exocyst complex (Koch et al. 2026). However, the results revealed that the TET8 interactome was enriched in proteins implicated in ER processes and Golgi vesicle transport, while putative cytoskeleton and actin-related proteins, such as myosin XI-K, were overrepresented in the PEN1 interactome, hinting at distinct biogenesis pathways for the 2 subpopulations of EVs.

Several VAMP-ASSOCIATED PROTEINS (VAPs) were identified in the proximity-labeling experiment as uniquely interacting with TET8. To further investigate the role of VAPs in EV biogenesis, the authors coexpressed VAP27 with TET8 in *N. benthamiana* and observed co-localization in ER punctate structures. By contrast, VAP27 did not co-localize with PEN1 in the ER. Furthermore, *VAP27* knockdown in *Arabidopsis thaliana* partially suppressed secretion of TET8+ EVs, but not PEN1+ EVs. Collectively, these data implicate VAP27 in the biogenesis of TET8+, but not PEN1+, EVs (Fig. 1).

**Figure 1:**
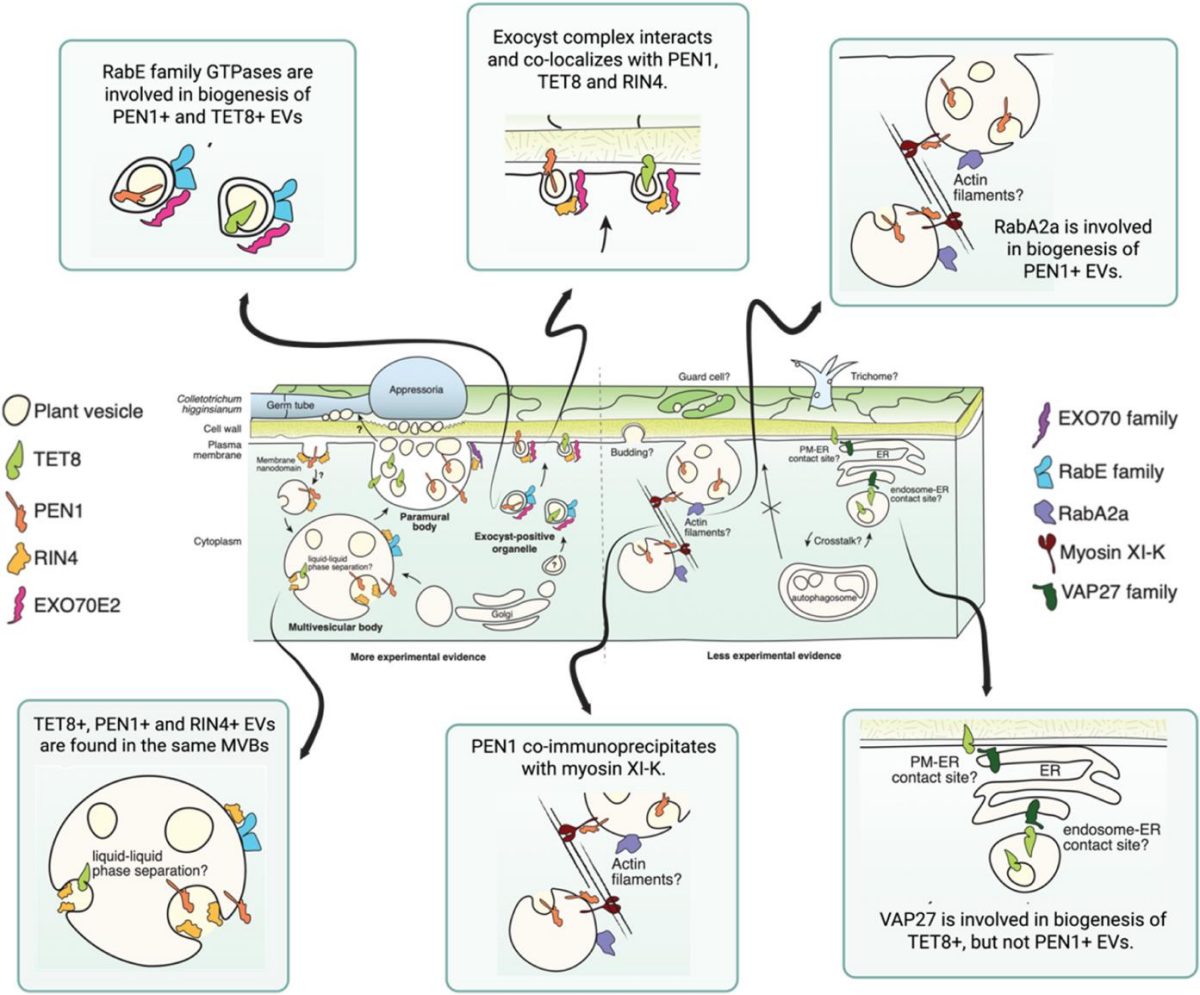
Model of the biogenesis pathways leading to EV secretion in Arabidopsis plants (adapted from Fig. 12, Koch et al. 2026. Created in BioRender. Teixeira, M. [2026] https://BioRender.com/v3hzg7l).

Rab GTPases are secreted with Arabidopsis EVs (Rutter and Innes 2017; Koch et al. 2025), and RabA2a has been shown to interact with PEN1 (Pang et al. 2022). The authors used a dominant negative allele of RabA2a to explore its role in secretion of EV subpopulations and found that disruption of RabA2a function led to a reduction in PEN1+ EV secretion but an increase in TET8+ EV secretion. By contrast, mutant analysis indicated that RabE family GTPases are involved in biogenesis of both TET8+ and PEN1+ EVs, suggesting distinct roles for different Rab GTPases in secretion of EV subpopulations (Fig. 1).

While RIN4 is a well-known regulator of plant immunity, its precise molecular function remains unclear. The authors showed that an Arabidopsis *rin4* mutant secreted fewer PEN1+ and TET8+ EVs than a plant that did not carry this mutation, implicating RIN4 in EV secretion and hinting at a mechanism through which RIN4 could contribute to plant defense.

The octameric exocyst complex mediates tethering and membrane fusion of secretory vesicles. The 70-kDa EXO70 subunit is responsible for targeting the exocyst complex to the membrane, and multiple EXO70 paralogs are present in plants. Notably, several EXO70 proteins contribute to pathogen resistance and are hijacked by pathogens to promote infection (De la Concepcion 2023). Members of the EXO70 family were among the exocyst proteins identified as candidate interactors of both PEN1 and TET8, and EXO70B2 was previously shown to co-localize with PEN1 in puncta near fungal haustoria (Ortmannová et al. 2022). Koch et al. demonstrated that EXO70B2 co-localizes with TET8 in cytoplasmic puncta and found that EXO70B2 associates with both PEN1 and TET8 in co-immunoprecipitation (Koch et al. 2026). The authors further probed the role of EXO70 proteins using an array of Arabidopsis lines with mutations in different members of the EXO70 family. Most mutant lines secreted fewer EVs overall, and most of these were also depleted in PEN1+ and TET8+ EV subpopulations. The strongest depletion in PEN1+ and TET8+ EVs was observed for *exo70e1* and *exo70e2* mutants. Disease assays revealed that *exo70* mutants are also more susceptible to the fungal pathogen *Colletotrichum higginsianum*, with the *exo70e1* and *exo70e2* mutants being the most susceptible, correlating with the effect of these mutations on PEN1+ and TET8+ EV secretion (Koch et al. 2026).

In summary, Koch et al. provide compelling evidence for distinct biogenesis pathways for TET8+ and PEN1+ EVs and identify some of the molecular players involved in secretion of these EV subpopulations. Intriguingly, the authors observed that fluorescent protein-tagged TET8 and PEN1 can co-localize to the same MVB-like structures. This requires further investigation, as both proteins were expressed under a 35S promoter and there were clear differences in the abundance of TET8 and PEN1 in MVB-like structures. However, this observation hints that ILVs originating from distinct biogenesis pathways may be found within the same MVB (Fig. 1).

The authors also present additional observations that open new avenues for investigation. For example, they observed that mutation of the MYB transcription factor GL1, known to function in trichome development, significantly reduces EV secretion. This suggests a link between GL1, EVs, and trichomes that merits further study. Additionally, because they note that PEN1+ EVs are abundant in guard cells, they speculate that different cell types may be specialized in secretion of specific EV subpopulations. Finally, Koch et al. report that mutating autophagy protein 5 (*atg5*) reduced TET8+ EV secretion, but the mechanism underpinning this remains unclear because the authors showed that TET8 does not co-localize with ATG8a. Given the importance of these tiny structures in diverse biological processes, understanding the formation and secretion of EV subpopulations may offer exciting new insights into plant physiology.

## Recent related articles in *Plant Physiology*:

- Kankaanpää et al. (Kankaanpää et al. 2024) reported that extracellular vesicles from Norway spruce are involved in transporting enzymes and precursors required for lignin formation.
- Chaya et al. (Chaya et al. 2024) characterized the extracellular vesicle proteomes of *Sorghum bicolor*.
- Rutter and Innes (Rutter and Innes 2017) provided the first, to our knowledge, demonstration of the isolation of extracellular vesicles from plants.

## Data Availability

No data is generated in this study.
